# The B7-H3/CD3 immune phenotype identifies prognostic subgroups in neuroblastoma

**DOI:** 10.3389/fimmu.2026.1829359

**Published:** 2026-05-18

**Authors:** Petar Rasic, Gordana Samardzija, Slavisa M. Djuricic, Nemanja Mitrovic, Ana Bogosavljevic, Andjela Milicevic, Ivan Soldatovic, Olivera Stefanovic, Nada Krstovski, Milos Kuzmanovic, Dragana Aleksic, Danka Redzic, Predrag Rodic, Jelena Lazic, Goran Milosevic, Jovana Svorcan, Ratko Radeta, Maja Milickovic, Dusko Dundjerovic

**Affiliations:** 1 Department of Abdominal Surgery, Mother and Child Health Care Institute of Serbia “Dr. Vukan Cupic”, Belgrade, Serbia; 2 Faculty of Medicine, University of Belgrade, Belgrade, Serbia; 3 Department of Clinical Pathology, Mother and Child Health Care Institute of Serbia “Dr. Vukan Cupic”, Belgrade, Serbia; 4 Faculty of Medicine, University of Banja Luka, Banja Luka, Bosnia and Herzegovina; 5 Department of Pathology, Clinic for Digestive Surgery, University Clinical Centre of Serbia, Belgrade, Serbia; 6 Department of Pathology, Institute for Oncology and Radiology of Serbia, Belgrade, Serbia; 7 Institute of Medical Statistics and Informatics, Faculty of Medicine, University of Belgrade, Belgrade, Serbia; 8 Department of Hematology and Oncology, University Children’s Hospital, Belgrade, Serbia; 9 Department of Hematology and Oncology, Mother and Child Health Care Institute of Serbia “Dr. Vukan Cupic”, Belgrade, Serbia; 10 Institute of Pathology, Faculty of Medicine, University of Belgrade, Belgrade, Serbia

**Keywords:** B7-H3, CD3, CD8, immune cell infiltration, immune phenotype, neuroblastoma, prognosis, survival

## Abstract

**Introduction:**

B7-H3 is an immune checkpoint molecule implicated in tumor immune evasion and adverse prognosis in multiple malignancies. This study aimed to characterize the relationships between tumor-cell B7-H3 expression, immune-cell infiltration, clinicopathological features, and survival in malignant neuroblastic tumors, and to define an immune phenotype with potential translational relevance.

**Methods:**

We retrospectively analyzed a population-representative cohort of 81 patients with neuroblastoma or nodular ganglioneuroblastoma diagnosed between 2009 and 2020. B7-H3 expression and CD3^+^, CD4^+^, CD8^+^, CD20^+^, and CD68^+^ immune-cell infiltration were evaluated by immunohistochemistry.

**Results:**

Tumors with high B7-H3 expression demonstrated reduced CD8^+^ T-cell infiltration (p = 0.045). High B7-H3 expression and low CD3^+^ T-cell infiltration were each associated with worse 60-month overall survival (OS; p = 0.025 and p = 0.014, respectively) and event-free survival (EFS; p = 0.047 and p = 0.019, respectively); however, after multivariable adjustment, only CD3^+^ T-cell infiltration retained prognostic significance. In a combined four-group model integrating these two markers, the B7-H3 low/CD3 high phenotype showed the most favorable prognosis, whereas B7-H3 high/CD3 low tumors had a 14-fold higher hazard of death (p = 0.014) and a 5.79-fold higher hazard of an EFS event (p = 0.010) compared with the favorable phenotype. Although the B7-H3/CD3 model showed only a trend toward improved OS model fit versus CD3 alone (p = 0.080), B7-H3 significantly stratified OS within the CD3-high subgroup (p = 0.019), enabling more precise identification of a particularly favorable phenotype. In a binary model, the favorable B7-H3 low/CD3 high phenotype was associated with significantly improved OS (HR = 0.095; p = 0.022) and EFS (HR = 0.219; p = 0.014) compared with all other phenotypes combined, and this effect remained significant after adjustment for age, *MYCN* status, and prognostic risk group.

**Conclusion:**

The B7-H3 low/CD3 high immune phenotype identifies patients with markedly favorable survival in malignant neuroblastic tumors and retains a protective association after adjustment for established clinical prognostic factors. However, given the limited statistical power of the current cohort, the overall incremental prognostic value of combined B7-H3/CD3 phenotyping requires evaluation in larger, independent cohorts.

## Introduction

Neuroblastoma (NB), together with ganglioneuroblastoma (GNB), is the most common extracranial pediatric solid tumor, with an estimated average annual incidence of approximately 12 cases per 1,000,000 children aged 0–14 years across European countries (1). Although formally classified as a rare disease (2), NB accounts for 8–10% of all childhood cancers and 15% of pediatric cancer-related mortality (3). Current management of NB relies on a risk-adapted approach, and advances in molecular diagnostics have enabled improved patient stratification and more precise treatment strategies (4–6). However, despite molecularly driven frameworks and the evolution of multimodal therapy, survival in patients with high-risk disease remains suboptimal, with rates of around 50% (3, 7, 8).

Ongoing research aimed at improving NB treatment is directed toward identifying novel molecular prognostic biomarkers and developing innovative therapeutic strategies (7, 9). Despite substantial improvements in immunotherapy for adult malignancies, the relatively low immunogenicity of pediatric solid tumors, and particularly NB, poses significant challenges for the development of effective immune-based therapies. A low tumor mutational burden and limited neoepitope expression in these neoplasms reduce the availability of targetable antigens. Additionally, these tumors are characterized by low MHC-I expression, which contributes to reduced T-cell infiltration (7–9). Therefore, further investigation of the factors shaping antitumor immune responses in NB is warranted, with particular emphasis on their prognostic and therapeutic implications (8, 9).

B7 homolog 3 (B7-H3) is an immune checkpoint molecule overexpressed in a wide range of malignant tumors, whereas its expression in healthy tissues is low or absent. This member of the B7 molecular family has been shown to modulate antitumor immunity by influencing the activity of multiple immune cell types, including CD4^+^ and CD8^+^ T cells, γδ T cells, CD45RO^+^ memory T cells, regulatory T cells, as well as macrophages and natural killer (NK) cells (10). Although the impact of B7-H3 on antitumor immunity remains a subject of debate, with studies reporting both inhibitory and stimulatory effects, most available evidence supports its predominantly suppressive role in T-cell–mediated immune responses (9–11). In addition, B7-H3 contributes to cancer progression through various non-immunological mechanisms, including the promotion of invasiveness, metastasis, and therapy resistance (9, 11). Accordingly, B7-H3 expression is associated with poor prognosis in multiple malignancies (12). Although B7-H3 expression in NB has been reported in several studies (13–18), only a limited number have specifically investigated its link with antitumor immunity and prognosis, suggesting an association with adverse outcomes (13, 14, 17, 18). Notably, the relationship between B7-H3 expression and tumor-infiltrating immune cells within the tumor microenvironment, as well as its relevance as an independent prognostic marker in NB, remains incompletely elucidated (9).

This population-representative study aimed to investigate the association of B7-H3 expression in NB and nodular GNB (GNB-N) tumor cells with clinicopathological characteristics and immune cell infiltration within the tumor microenvironment, including CD3^+^, CD4^+^, CD8^+^, and CD20^+^ lymphocytes, as well as CD68^+^ macrophages. We further evaluated the individual prognostic relevance of B7-H3 expression and immune cell–related markers as independent variables and investigated whether their integration into immune phenotypes provides additional prognostic stratification, thereby delineating subgroups with potential biological and clinical significance.

## Materials and methods

### Patients

**Figure 1:**
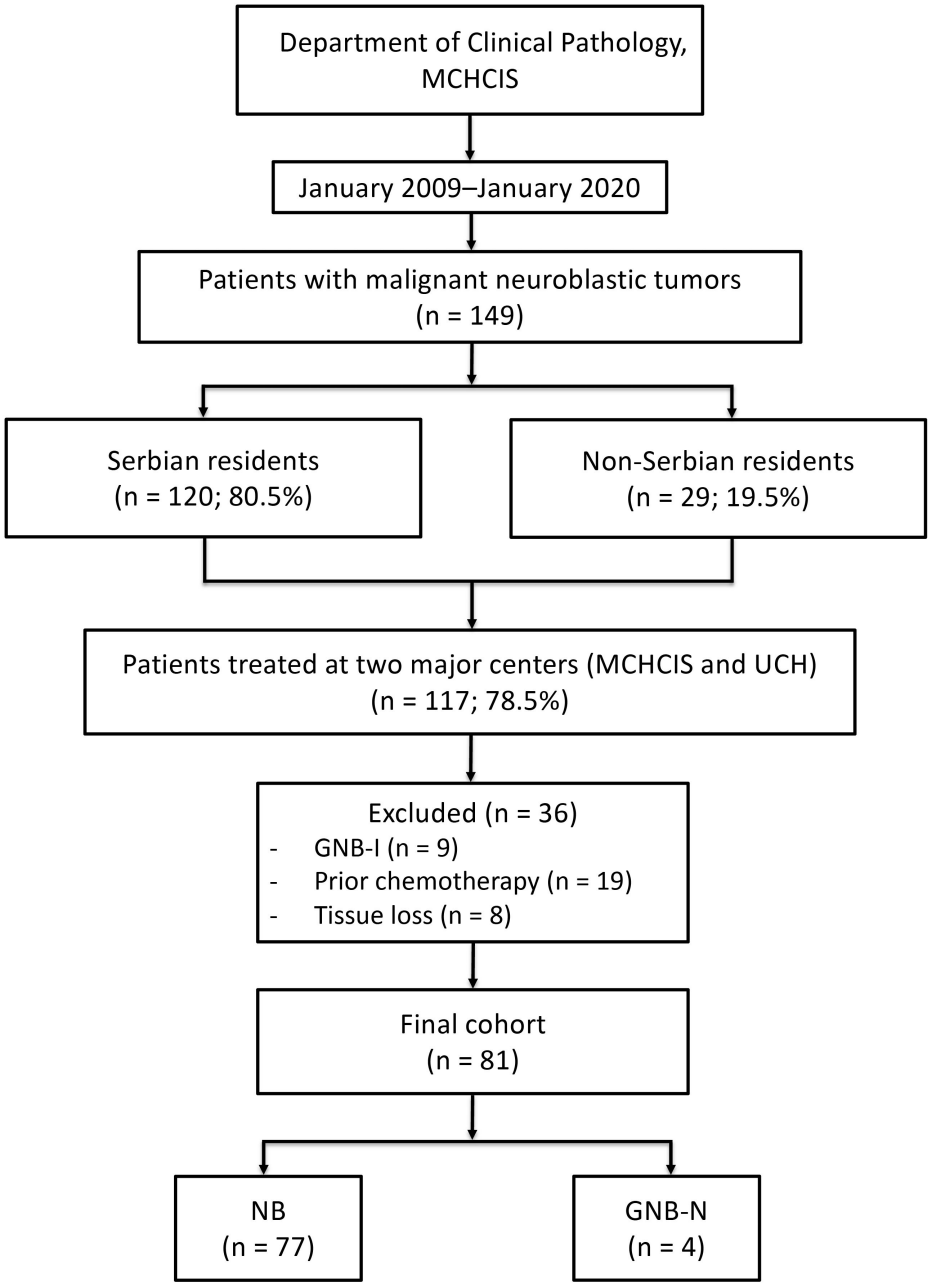
Patient selection flowchart. NB, neuroblastoma; GNB-N, nodular ganglioneuroblastoma; GNB-I, intermixed ganglioneuroblastoma; MCHCIS, Mother and Child Health Care Institute of Serbia; UCH, University Children’s Hospital.

This retrospective study included patients with NB and GNB-N whose histopathological diagnoses were established between January 2009 and January 2020 at the Department of Clinical Pathology of the Mother and Child Health Care Institute of Serbia (MCHCIS) “Dr. Vukan Cupic”, which serves as the national reference laboratory for neuroblastic tumors. Tumor tissue used for diagnosis was obtained during treatment at two tertiary referral pediatric centers: the MCHCIS and the University Children’s Hospital (UCH), Belgrade, Serbia. Cases with GNB-intermixed were excluded because the neuroblastic component of these tumors is characterized by microscopic nests of neuroblastic cells randomly distributed within a Schwannian stroma-rich, ganglioneuromatous background—an architectural pattern that may limit reliable assessment of tumor-cell B7-H3 expression. In addition, GNB-intermixed represents a biologically distinct entity with clinical behavior differing from that of NB, potentially introducing additional variability and confounding the interpretation of prognostic analyses. In contrast, in GNB-N, the neuroblastic component is organized into one or more distinct nodules, allowing reliable evaluation of tumor-cell B7-H3 expression. Moreover, these tumors exhibit clinical behavior similar to NB, determined by the characteristics of the neuroblastic nodules. Patients who had received chemotherapy before tissue sampling were also excluded, as treatment-related changes may alter tumor morphology and immune cell infiltration and, according to the International Neuroblastoma Pathology Classification (INPC), preclude reliable assessment of histopathological prognostic features (19). Finally, cases with non-representative histopathological material due to tissue loss during processing were excluded. Patient identification and study population are shown in **Figure 1**. The study was approved by the Ethics Committees of MCHIS (approval No. 8/49) and UCH (approval No. 16/157) and was conducted in accordance with the principles of the Declaration of Helsinki. Given the retrospective design of the study, both Ethics Committees concluded that informed consent obtained for diagnostic and treatment procedures during initial management was sufficient and therefore waived the requirement for additional informed consent (waiver No. 8/135 and No. 16/293, respectively).

Clinical data were retrospectively collected from institutional medical records at the MCHCIS and the UCH. Most patients included in this study were staged according to the International Neuroblastoma Risk Group Staging System (INRGSS) (20) during initial treatment, while the remaining cases were staged based on the International Neuroblastoma Staging System (INSS) (21). To ensure a uniform staging framework across the entire cohort and enable consistent analysis of B7-H3 expression within patient subgroups, retrograde restaging was performed, whereby patients originally staged according to INSS were subsequently reassigned to INRGSS categories.

Patients were diagnosed and treated according to contemporary risk-adapted protocols (22–28), with all but two managed using International Society of Paediatric Oncology Europe Neuroblastoma Group (SIOPEN)-aligned protocols. High-risk patients received therapy based on the High Risk Neuroblastoma Study 1 (HR-NBL1) (22) protocol (n = 23). Low- and intermediate-risk patients were managed according to the Low and Intermediate Risk Neuroblastoma European Study (LINES) (23) protocol (n = 26) or protocols broadly consistent with the LINES framework, including the Infant Neuroblastoma European Study (INES) (24) (n = 7), and the SIOPEN study for treatment of children over the age of one year with unresectable localised neuroblastoma without *MYCN* amplification (n = 7) (25). Of note, three patients classified at diagnosis as INRG stage L2, aged >18 months, with unfavorable histology received HR-NBL1–based therapy following a multidisciplinary team decision, in line with practices reported by some cooperative groups (6). Sixteen patients with INRG L1, *MYCN* non-amplified disease were managed with observation alone following complete tumor resection, in accordance with the Localized Neuroblastoma European Study Group 2 (LNESG2) protocol (26), consistent with the LINES framework (23). Two patients underwent a comprehensive diagnostic work-up and primary tumor resection at the MCHCIS, where the diagnosis of NB was histopathologically confirmed, and the surgical tissue obtained during this procedure was used for the present study. Both patients subsequently continued oncologic treatment for high-risk NB at St. Jude Children’s Research Hospital according to the Children’s Oncology Group (COG) ANBL17P1 (27) and NB2012 (28) protocols. Information regarding the entire course of treatment of these patients was obtained from follow-up medical documentation available at the MCHCIS. St. Jude Children’s Research Hospital had no direct involvement in the present study.

To ensure uniformity of prognostic risk classification, patients were stratified according to the SIOPEN High-Risk Neuroblastoma Standard Clinical Practice Guidelines (22). High-risk disease was defined as INRG stage M tumors diagnosed in patients older than 365 days, regardless of *MYCN* status, or any INRG stage L2, M, or Ms tumors with *MYCN* amplification at any age. All remaining patients were classified as non–high-risk.

Survival was assessed over a fixed 60-month follow-up period for each patient, and no patients were lost to follow-up. Overall survival (OS) was calculated as the time from diagnosis to death or to the end of follow-up if death did not occur. Event-free survival (EFS) was defined as the interval from diagnosis to the first occurrence of an event (relapse, progression, or death) or to the end of follow-up in patients without an event. Progression was defined as the appearance of any new lesion, a >25% increase in any measurable lesion, or newly positive bone marrow following a previously negative assessment (21, 29).

The histopathological diagnosis was established according to the INPC (19). Data on the histopathological characteristics of the tumors were obtained from original pathology reports and from a re-evaluation of histological specimens performed by two experienced pathologists, confirming the initial findings. These data included INPC histological category, degree of tumor differentiation, mitosis–karyorrhexis index (MKI), and INPC prognostic group. In cases of GNB-N, these parameters were determined based on the characteristics of the NB nodule.

### Histopathological analysis

#### Tissue samples

Tumor tissue samples were obtained from formalin-fixed, paraffin-embedded (FFPE) specimens archived at the Department of Clinical Pathology, MCHCIS. For each patient, one representative FFPE block was selected based on minimal tumor necrosis, minimal intratumoral hemorrhage, and maximal immune-cell infiltration. The selected FFPE blocks were sectioned on a rotary microtome into 4 µm-thick sections, mounted on standard glass microscope slides, and dried at 60 °C for 30 minutes. After drying, the samples were subjected to routine hematoxylin and eosin (H&E) staining using an automated slide staining system (Automated Slide Stainer SS-30, Myr, Spain).

#### Immunohistochemistry

**Figure 2:**
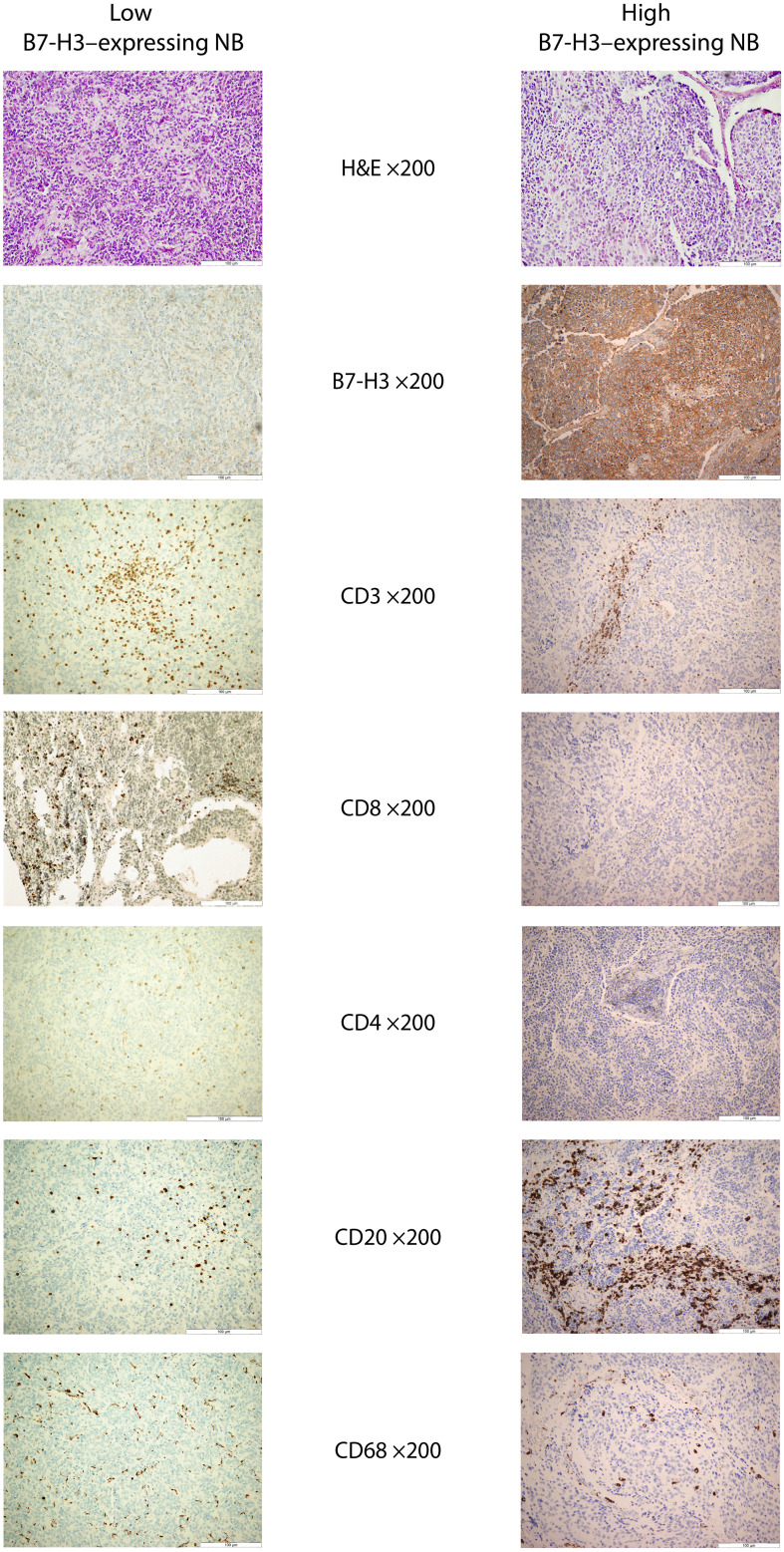
Representative histological and immunohistochemical features of low- and high-B7-H3–expressing neuroblastomas. Representative photomicrographs illustrate the inverse association between tumor-cell B7-H3 expression and CD8^+^ T-cell infiltration. Images include hematoxylin and eosin (H&E) staining and immunohistochemical staining for B7-H3, CD3, CD4, CD8, CD20, and CD68 (all ×200 magnification). B7-H3 staining shows membranous and cytoplasmic expression in tumor cells. CD3 marks total tumor-infiltrating T lymphocytes; CD4 and CD8 identify T-cell subsets; CD20 highlights B lymphocytes; and CD68 identifies macrophages. Abbreviations: B7-H3, B7 homolog 3; H&E, hematoxylin and eosin; NB, neuroblastoma.

Immunohistochemical staining was performed using an automated slide staining system (Autostainer Link 48, Dako, Agilent Technologies). The following antibodies were used: B7-H3 (clone RBT-B7H3, BioSB, Ready-to-use), CD3 (polyclonal, Dako, Ready-to-use), CD4 (clone 4B12, Dako, Ready-to-use), CD8 (clone 4B11, Novocastra, 1:50), CD20 (clone L26, Dako, Ready-to-use), and CD68 (clone PG-M1, Dako, Ready-to-use) (**Figure 2**).

All microscopic analyses were performed using an Olympus BX43 microscope (Olympus Corporation, Tokyo, Japan). B7-H3 expression in tumor cells was independently assessed by two experienced pathologists who were not involved in the initial tissue sample re-evaluation or immunohistochemical staining and were fully blinded to all clinical and outcome data at the time of evaluation. Tumor-cell B7-H3 expression was evaluated using the H-score method as previously described: H-score = (% of unstained tumor cells × 0) + (% of weakly stained tumor cells × 1) + (% of moderately stained tumor cells × 2) + (% of strongly stained tumor cells × 3) (30). In cases of discrepant scoring, a consensus H-score was established following joint review and used for all subsequent analyses. The optimal cut-off value for B7-H3 expression was determined using a data-driven approach based on the continuous consensus H-score values and applied to stratify cases into low- and high-expression groups. Inter-observer agreement was assessed using Cohen’s kappa coefficient, based on the initial independent (pre-consensus) assessments of both pathologists after application of this cut-off.

The density of CD3^+^, CD4^+^, and CD8^+^ T cells, CD20^+^ B cells, and CD68^+^ macrophages within the tumor area was quantified using QuPath software (version 0.5.1) (31). Digital images were acquired using an Olympus DP70 camera (Olympus Corporation, Tokyo, Japan) mounted on an Olympus BX43 microscope and imported into the software for analysis. For each case, representative regions of interest were manually annotated within the tumor area, which was defined as the area occupied by tumor cells, including the associated intratumoral and peritumoral stroma. In line with previously published methodology (30, 32), five fields within the tumor area with the most intense inflammatory infiltrate were selected at low magnification (×40), and immune cells were quantified at high-power magnification (×200). Mean values from the five regions were calculated, yielding a representative immune cell density for each tumor. This approach was selected to account for the heterogeneous and spatially compartmentalized distribution of immune cells in malignant neuroblastic tumors (8, 33–35). A separate QuPath project was created for each stain, and detection and quantification were performed in batches using QuPath’s built-in Positive Cell Detection algorithm, based on nuclear segmentation and 3,3′-diaminobenzidine (DAB) staining intensity. Detection parameters, including background correction, staining thresholds, minimum and maximum cell area, and nuclear inclusion settings, were initially optimized on training images for each stain and subsequently refined to generate a project-specific script for each antibody (36–38). The results were exported as a structured XLSX file.

### Statistical analysis

Categorical clinicopathological variables were summarized using counts and percentages. For the extent of immune cell infiltration, median values of the per-tumor cell densities were reported and used to dichotomize infiltration into high and low groups. Associations between categorical variables were analyzed using the χ² test, including χ² test for trend where appropriate, Fisher’s exact test, and Fisher–Freeman–Halton test for multi-category variables. Survival outcomes, including 60-month OS and EFS, were estimated using the Kaplan–Meier method and compared using the log-rank test. Cox proportional hazards regression was used to evaluate associations between variables and survival outcomes in univariable and multivariable models. In the CD3-high subgroup, where the number of events was particularly low, Firth-penalized Cox regression was applied to reduce small-sample bias and improve the stability of effect estimates. Model discrimination was assessed using Harrell’s concordance index (C-index), and model fit was compared using the likelihood ratio test (LRT). No formal multiple testing correction was applied, as the analyses were based on a limited number of pre-specified, biologically driven hypotheses.

The optimal cut-off value for tumor-cell B7-H3 expression was determined using a minimum p-value approach based on log-rank statistics implemented in X-tile software (39). The selected cut-off was further assessed using receiver operating characteristic (ROC) curve analysis.

Statistical analyses were performed using SPSS Statistics version 30.0 (IBM Corp., Armonk, NY, USA), except for minimum p-value cut-off determination in X-tile and selected analyses conducted in R software version 4.5.2 (R Foundation for Statistical Computing, Vienna, Austria). In R, Firth-penalized Cox regression was performed using the *coxphf* package. Model discrimination analyses, including estimation of Harrell’s C-index and LRT–based model comparisons, were conducted using the *survival* package. All statistical tests were two-sided, and p-values < 0.05 were considered statistically significant.

## Results

### Study population and patient characteristics

An initial search of the archive of the Department of Clinical Pathology at the MCHCIS identified 149 patients with malignant neuroblastic tumors treated at Serbian medical centers during the study period. Of these, 120 patients (80.5%) were residents of Serbia, while 29 patients (19.5%) originated from neighboring countries, including Montenegro and Bosnia and Herzegovina. Among Serbian residents, this corresponded to an average of 10.9 newly diagnosed cases per year. Among all patients treated in Serbian centers, 117 patients (78.5%) were managed at the two largest tertiary referral institutions: the MCHCIS and the UCH. After application of the predefined inclusion and exclusion criteria, 81 patients were included in the final cohort, comprising 77 cases of NB (95.1%) and 4 cases of GNB-N (4.9%) (**Figure 1**). Over the 60-month follow-up period, 17 deaths and 23 EFS events were observed. Baseline clinicopathological characteristics of the study cohort are summarized in **Table 1**.

**Table 1:** Clinicopathological characteristics and their association with tumor-cell B7-H3 expression in patients with malignant neuroblastic tumors.

Clinical parameter	Cases	Low B7-H3 (≤ 200)	High B7-H3 (> 200)	p-value
Sex				0.753 ^a^
Male	40	25 (62.5%)	15 (37.5%)	
Female	41	27 (65.9%)	14 (34.1%)	
Age				0.325 ^a^
<18 months	45	31 (68.9%)	14 (31.1%)	
≥18 months	36	21 (58.3%)	15 (41.7%)	
Primary tumor site				0.403 ^b^
Adrenal gland	49	33 (67.3%)	16 (32.7%)	
Retroperitoneum (Extra-adrenal)	23	15 (65.2%)	8 (34.8%)	
Mediastinum	3	2 (66.7%)	1 (33.3%)	
Pelvis	4	2 (50.0%)	2 (50.0%)	
Neck	2	0 (0.0%)	2 (100.0%)	
INRG stage				0.527 ^b^
L1	21	15 (71.4%)	6 (28.6%)	
L2	24	15 (62.5%)	9 (37.5%)	
M	21	11 (52.4%)	10 (47.6%)	
Ms	15	11 (73.3%)	4 (26.7%)	
Prognostic risk				0.269 ^a^
Non-high	59	40 (67.8%)	19 (32.2%)	
High	22	12 (54.5%)	10 (45.5%)	
INPC histological category				0.615 ^a^
NB	77	50 (64.9%)	27 (35.1%)	
GNB-N	4	2 (50.0%)	2 (50.0%)	
Degree of tumor differentiation				0.800 ^c^
Undifferentiated	12	7 (58.3%)	5 (41.7%)	
Poorly differentiated	64	42 (65.6%)	22 (34.4%)	
Differentiating	5	3 (60.0%)	2 (40.0%)	
MKI				0.242 ^c^
Low	28	21 (75.0%)	7 (25.0%)	
Intermediate	38	21 (55.3%)	17 (44.7%)	
High	13	8 (61.5%)	5 (38.5%)	
N/A	2	N/A	N/A	
INPC prognosis				0.981 ^a^
Favorable	38	24 (63.2%)	14 (36.8%)	
Unfavorable	41	26 (63.4%)	15 (36.6%)	
N/A	2	N/A	N/A	
*MYCN* status				0.270 ^d^
Non-amplified	72	48 (66.7%)	24 (33.3%)	
Amplified	9	4 (44.4%)	5 (55.6%)	

Patients were stratified according to B7-H3 expression using an H-score cutoff of 200 (low ≤ 200 vs high > 200). B7-H3, B7 homolog 3; GNB-N, nodular ganglioneuroblastoma; INPC, International Neuroblastoma Pathology Classification; INRG, International Neuroblastoma Risk Group; MKI, mitosis–karyorrhexis index; N/A, not available; NB, neuroblastoma. χ² tests (a), Fisher–Freeman–Halton tests for multi-category variables (b), χ² test for linear trend across ordered categories (c), and Fisher’s Exact Test (d).

### Association of B7-H3 expression with clinicopathological features and prognosis

Tumor-cell B7-H3 expression was detected in all tumor samples, with a median H-score of 170 (range: 10–300). Based on H-score values, patients were stratified into low and high B7-H3 expression groups. Since there was no established cut-off for B7-H3 expression in the literature, we determined the optimal B7-H3 H-score threshold using two approaches described in previous studies. First, we applied the minimum p-value (maximally selected log-rank) method for OS and EFS (40, 41). As a complementary approach, we performed ROC curve analysis for the same endpoints and identified the optimal cut-off using the Youden index (42). All four analyses converged on the same separation point and defined low B7-H3 expression as H-score ≤200 and high B7-H3 expression as H-score >200. Inter-observer agreement for B7-H3 scoring was high, with a Cohen’s kappa value of 0.82, indicating almost perfect agreement.

High B7-H3 expression was detected in 29/81 (35.8%) patients. There was no difference in B7-H3 expression regarding sex, age, primary tumor site, INRG stage, prognostic risk group, INPC histological category, degree of tumor differentiation, MKI, INPC prognostic group, and *MYCN* status (**Table 1**).

**Figure 3:**
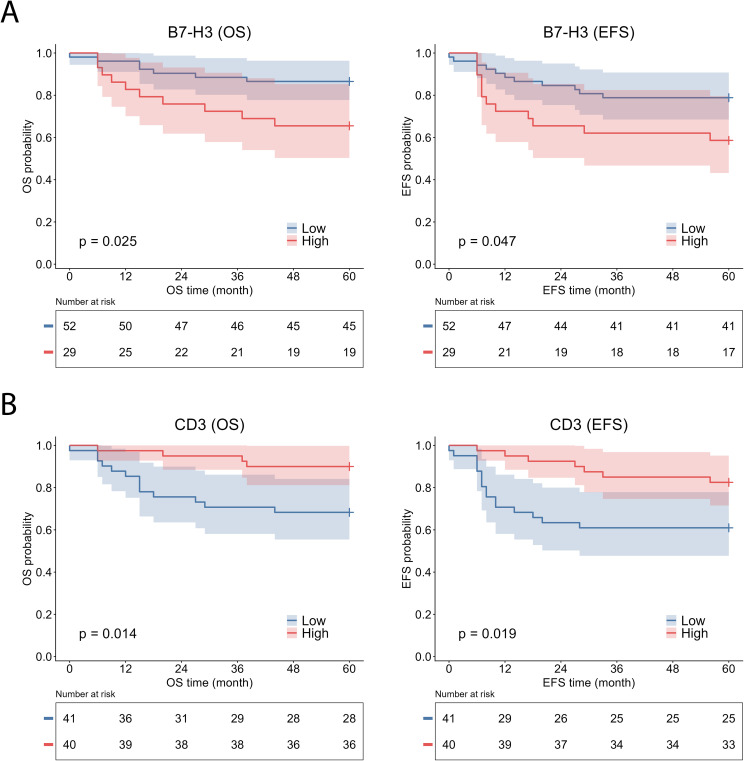
Kaplan–Meier survival analysis according to tumor-cell B7-H3 expression and CD3^+^ T-cell infiltration in malignant neuroblastic tumors. Kaplan–Meier curves show overall survival (OS) and event-free survival (EFS) stratified by B7-H3 expression **(A)** and CD3^+^ T-cell infiltration **(B)**. High and low B7-H3 expression were defined as an H-score >200 and ≤200, respectively. CD3^+^ T-cell infiltration was dichotomized into high and low groups based on median values. B7-H3, B7 homolog 3; EFS, event-free survival; OS, overall survival.

Kaplan–Meier survival analysis demonstrated that high B7-H3 expression was associated with significantly worse OS (log-rank χ² = 5.035, p = 0.025) and EFS (log-rank χ² = 3.935, p = 0.047) (**Figure 3A**). In univariable Cox regression analysis, high B7-H3 expression was significantly associated with inferior OS and showed a trend toward worse EFS (**Table 2**).

**Table 2:** Univariable and multivariable cox regression analyses of tumor-cell B7-H3 expression and survival outcomes in malignant neuroblastic tumors.

Endpoint	Model	Variable	HR	95% CI	p-value
OS	Univariable	B7-H3 high	2.87	1.09–7.53	**0.033**
	Multivariable (age-adjusted)	B7-H3 high	2.52	0.96–6.63	0.062
		Age ≥18 months	10.39	2.37–45.62	**0.002**
	Multivariable (*MYCN*-adjusted)	B7-H3 high	2.52	0.95–6.72	0.064
		*MYCN* amplification	3.79	1.31–10.99	**0.014**
	Multivariable (prognostic risk–adjusted)	B7-H3 high	2.23	0.84–5.90	0.107
		High risk	11.05	3.55–34.37	**<0.001**
EFS	Univariable	B7-H3 high	2.23	0.98–5.06	0.055
	Multivariable (age-adjusted)	B7-H3 high	2.16	0.95–4.93	0.066
		Age ≥18 months	5.35	1.98–14.48	**0.001**
	Multivariable (*MYCN*-adjusted)	B7-H3 high	2.05	0.90–4.68	0.090
		*MYCN* amplification	3.52	1.37–9.08	**0.009**
	Multivariable (prognostic risk–adjusted)	B7-H3 high	1.91	0.83–4.35	0.126
		High risk	5.23	2.24–12.21	**<0.001**

Survival outcomes were assessed over a fixed 60-month follow-up period. Bold values indicate statistical significance (p < 0.05). B7-H3, B7 homolog 3; CI, confidence interval; EFS, event-free survival; HR, hazard ratio; OS, overall survival.

Given the limited number of events (n = 17 for OS and n = 23 for EFS), multivariable Cox regression analyses were intentionally restricted to parsimonious models that included B7-H3 and a single additional covariate per model, to minimize the risk of overfitting. After adjustment for age and *MYCN* status, both age ≥18 months and *MYCN* amplification remained independently associated with poorer OS and EFS, while high B7-H3 expression continued to show a consistent trend toward inferior survival without retaining independent statistical significance. In the prognostic risk–adjusted model, which incorporates disease stage, *MYCN* status, and age at diagnosis, high-risk disease status emerged as the strongest independent predictor of both OS and EFS, whereas B7-H3 expression was not independently associated with survival outcomes (**Table 2**).

### Association of B7-H3 expression with immune cell infiltration

We examined whether tumor-cell B7-H3 expression was associated with the degree of immune-cell infiltration (**Table 3**), using median-based dichotomization for each immune cell marker as described by previous studies (43–45). No significant associations were observed between B7-H3 expression and CD3^+^, CD4^+^, CD20^+^, or CD68^+^ cell densities, with similar distributions of low and high infiltration across B7-H3 subgroups. In contrast, CD8^+^ T-cell infiltration showed a statistically significant inverse association with B7-H3 expression (p = 0.045). Tumors with high B7-H3 expression exhibited lower CD8^+^ infiltration, whereas tumors with low B7-H3 expression more frequently displayed high CD8^+^ infiltration (**Figure 2**).

**Table 3:** Association between tumor-cell B7-H3 expression and immune cell infiltration in malignant neuroblastic tumors.

Immune marker	Cut-off (cells/HPF)	B7-H3 expression (H-score)		p-value
		Low (≤ 200)	High (> 200)	
CD3	> 831	29 (55.8%)	11 (37.9%)	0.124
CD4	> 166	24 (46.2%)	16 (55.2%)	0.436
CD8	> 70	30 (57.7%)	10 (34.5%)	**0.045**
CD20	> 233	28 (53.8%)	12 (41.4%)	0.282
CD68	> 249	27 (51.9%)	13 (44.8%)	0.540

Immune markers were dichotomized using the median as the cut-off value. p-values were calculated using the χ² test. Bold values indicate statistical significance (p < 0.05). B7-H3, B7 homolog 3; HPF, high-power field.

### Association of immune cell infiltration with prognosis

When evaluating the association of immune cell subsets with survival, CD3^+^ T-cell density demonstrated a consistent and statistically significant prognostic effect, with CD3-high tumors exhibiting superior outcomes compared with CD3-low cases for both OS (log-rank χ²=6.003, p=0.014) and EFS (log-rank χ²=5.513, p=0.019) (**Figure 3B**). These findings were confirmed by Cox proportional hazards regression analyses. In univariable analysis, high intratumoural CD3^+^ T-cell infiltration was significantly associated with nearly fourfold better OS and nearly threefold better EFS. This favorable prognostic effect remained independent in parsimonious multivariable models adjusted for age, *MYCN* status, and prognostic risk classification (**Table 4**).

**Table 4:** Univariable and multivariable cox regression analyses of intratumoral CD3^+^ T-cell infiltration and survival outcomes in malignant neuroblastic tumors.

Endpoint	Model	Variable	HR	95% CI	p-value
OS	Univariable	CD3 high	0.27	0.09–0.84	**0.023**
	Multivariable (age–adjusted)	CD3 high	0.30	0.10–0.92	**0.035**
		Age ≥18 months	10.46	2.38–45.93	**0.002**
	Multivariable (*MYCN*–adjusted)	CD3 high	0.30	0.10–0.91	**0.033**
		*MYCN* amplification	3.93	1.37–11.29	**0.011**
	Multivariable (prognostic risk–adjusted)	CD3 high	0.30	0.10–0.92	**0.035**
		High risk	11.61	3.75–36.00	**<0.001**
EFS	Univariable	CD3 high	0.36	0.15–0.88	**0.025**
	Multivariable (age–adjusted)	CD3 high	0.37	0.15–0.91	**0.030**
		Age ≥18 months	5.35	1.97–14.49	**0.001**
	Multivariable (*MYCN*–adjusted)	CD3 high	0.38	0.16–0.94	**0.036**
		*MYCN* amplification	3.55	1.38–9.11	**0.008**
	Multivariable (prognostic risk–adjusted)	CD3 high	0.39	0.16–0.94	**0.037**
		High risk	5.38	2.31–12.53	**<0.001**

Survival outcomes were assessed over a fixed 60-month follow-up period for each patient. Bold values indicate statistical significance (p < 0.05). CI, confidence interval; EFS, event-free survival; HR, hazard ratio; OS, overall survival.

**Figure 4:**
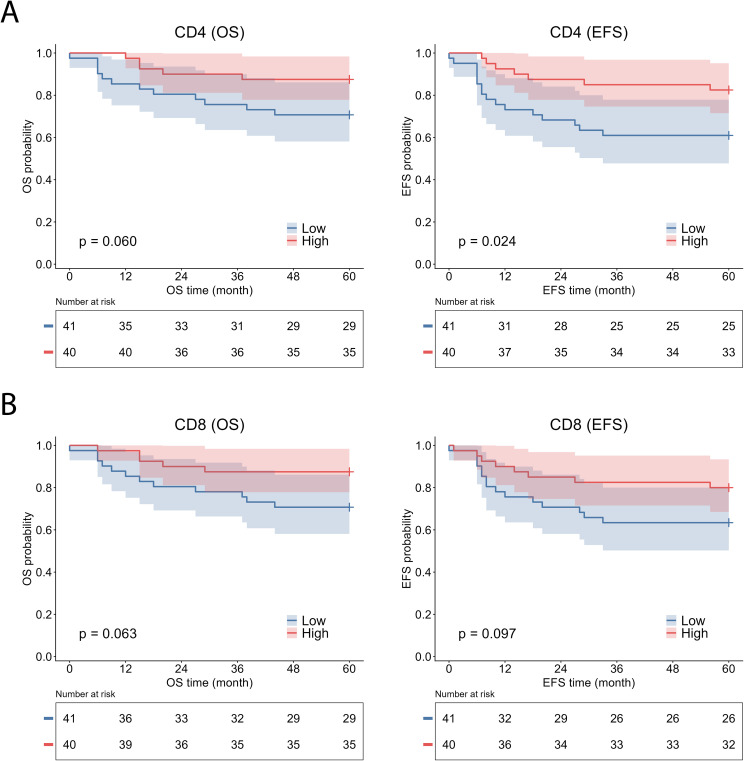
Kaplan–Meier survival analysis according to CD4^+^ and CD8^+^ T-cell infiltration in malignant neuroblastic tumors. Kaplan–Meier curves show overall survival (OS) and event-free survival (EFS) stratified by CD4^+^ **(A)** and CD8^+^ **(B)** T-cell infiltration. CD4⁺ and CD8⁺ T-cell infiltration were dichotomized into high and low groups based on median values. EFS, event-free survival; OS, overall survival.

**Figure 5:**
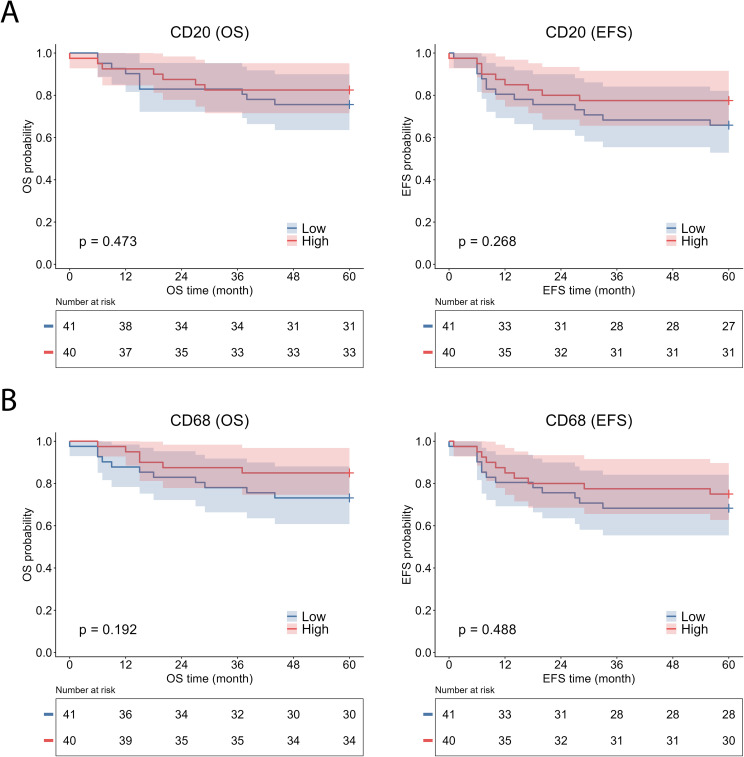
Kaplan–Meier survival analysis according to CD20^+^ B-cell and CD68^+^ macrophage infiltration in malignant neuroblastic tumors. Kaplan–Meier curves show overall survival (OS) and event-free survival (EFS) stratified by CD20^+^ B-cell infiltration **(A)** and CD68^+^ macrophage infiltration **(B)**. CD20⁺ B-cell and CD68⁺ macrophage infiltration were dichotomized into high and low groups based on median values. EFS, event-free survival; OS, overall survival.

CD4^+^ T-cell infiltration similarly favored better survival, reaching statistical significance for EFS (log-rank χ² = 5.085, p = 0.024) and demonstrating a trend toward improved OS that did not reach statistical significance (log-rank χ² = 3.550, p = 0.060) (**Figure 4A**). High CD8^+^ T-cell infiltration also showed a trend toward improved outcomes; however, statistical significance was not achieved (OS: log-rank χ² = 3.448, p = 0.063; EFS: log-rank χ² = 2.753, p = 0.097) (**Figure 4B**). Infiltration of CD20^+^ B cells and CD68^+^ macrophages showed no association with either survival endpoint (CD20: OS log-rank χ² = 0.515, p = 0.473; EFS log-rank χ² = 1.228, p = 0.268; CD68: OS log-rank χ² = 1.704, p = 0.192; EFS log-rank χ² = 0.480, p = 0.488) (**Figures 5A, B**).

### Combined prognostic impact of B7-H3 expression and immune cell infiltration

**Figure 6:**
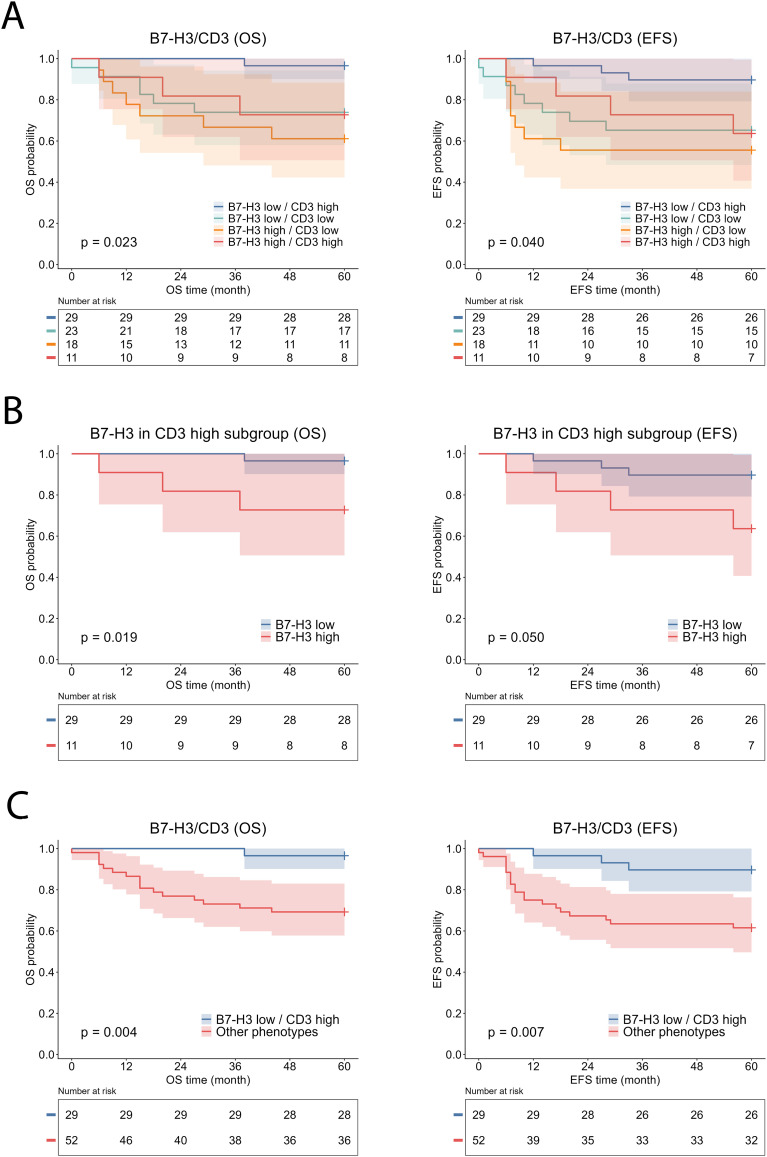
Kaplan–Meier survival analysis according to combined tumor-cell B7-H3 expression and CD3^+^ T-cell infiltration in malignant neuroblastic tumors. Kaplan–Meier curves show overall survival (OS) and event-free survival (EFS). Survival was first analyzed according to the combined B7-H3/CD3 immune phenotype using a four-group model **(A)**. Subsequently, survival was analyzed within the CD3-high subgroup according to B7-H3 expression **(B)**. Finally, survival was assessed using a simplified two-group model comparing the most favorable B7-H3 low/CD3 high phenotype with all other phenotypes combined **(C)**. High and low B7-H3 expression were defined as an H-score >200 and ≤200, respectively. CD3⁺ T-cell infiltration was dichotomized into high and low groups based on median values. B7-H3, B7 homolog 3; EFS, event-free survival; OS, overall survival.

We subsequently analyzed the combined effects of tumor-cell B7-H3 expression and CD3^+^ T-cell infiltration, the most prominent immune cell–based prognostic marker in our study. Patients were stratified into four immune phenotypes based on the combination of B7-H3 expression (low/high) and CD3^+^ T-cell infiltration (low/high). Kaplan–Meier analysis demonstrated significant separation of survival curves across the four immune phenotypes for both endpoints (OS log-rank χ² = 9.510, p = 0.023; EFS log-rank χ² = 8.312, p = 0.040) (**Figure 6A**). Among these, the B7-H3 low/CD3 high phenotype demonstrated the most favorable survival outcomes, with 60-month OS and EFS rates of 96.6% and 89.7%, respectively, and was therefore used as the reference group for subsequent analyses.

In contrast, patients with high B7-H3 expression and low CD3^+^ T-cell infiltration exhibited the poorest survival outcomes, with an approximately 14-fold higher hazard of death and a 5.79-fold higher hazard of an EFS event compared with the reference phenotype. The remaining immune phenotypes demonstrated intermediate survival patterns. Tumors characterized by low B7-H3 expression and low CD3^+^ cell infiltration were associated with significantly worse OS and EFS compared with the reference group, whereas the B7-H3 high/CD3 high phenotype showed a consistent trend toward poorer outcomes that did not reach statistical significance (**Table 5**).

**Table 5:** Prognostic impact of combined B7-H3/CD3 immune phenotypes on survival outcomes in malignant neuroblastic tumors.

B7-H3/CD3 phenotype	OS HR (95% CI)	p-value	EFS HR (95% CI)	p-value
Low/High (reference)	1.00	–	1.00	–
Low/Low	8.83 (1.06–73.38)	**0.044**	4.10 (1.09–15.63)	**0.037**
High/High	8.92 (0.93–85.82)	0.058	3.87 (0.87–17.70)	0.077
High/Low	14.00 (1.72–113.94)	**0.014**	5.79 (1.53–21.88)	**0.010**

The B7-H3 low/CD3 high immune phenotype was used as the reference category. p-values were calculated using cox proportional hazards regression. Bold values indicate statistical significance (p < 0.05). B7-H3, B7 homolog 3; CI, confidence interval; EFS, event-free survival; HR, hazard ratio; OS, overall survival.

### Model discrimination analysis

In model discrimination analysis for OS, the combined four-group B7-H3/CD3 classification demonstrated a higher Harrell’s C-index than CD3^+^ T-cell infiltration alone (0.704, 95% CI 0.603–0.805 vs. 0.654, 95% CI 0.552–0.756). LRT indicated a trend toward improved model fit for OS (χ² = 5.062, df = 2, p = 0.080), without reaching statistical significance. For EFS, the combined phenotyping likewise showed a higher C-index compared with CD3 alone (0.676, 95% CI 0.579–0.773 vs. 0.635, 95% CI 0.543–0.726); however, LRT did not demonstrate a statistically significant improvement in model fit (χ² = 3.644, df = 2, p = 0.162).

### Identification and prognostic value of the favorable phenotype

We further assessed whether B7-H3 expression provides additional prognostic stratification within the CD3-high subgroup and improves the identification of a favorable phenotype. Observed mortality was 10.0% (4/40) in the CD3-high group and 3.4% (1/29) in the B7-H3 low/CD3 high group, while EFS event rates were 17.5% (7/40) and 10.3% (3/29), respectively. Kaplan–Meier analysis in the CD3-high subgroup demonstrated significantly better OS in the B7-H3 low/CD3 high phenotype compared with the B7-H3 high/CD3 high phenotype (log-rank χ² = 5.488, p = 0.019). For EFS, a borderline improvement was observed in the B7-H3 low/CD3 high phenotype (log-rank χ² = 3.854, p = 0.050) (**Figure 6B**). Due to the limited number of events, Firth-penalized Cox regression was performed, demonstrating that, within the CD3-high subgroup, high B7-H3 expression was associated with significantly increased hazard of death (HR = 7.20, 95% CI 1.18–74.48, p = 0.033). A similar, though non-significant, trend was observed for EFS (HR = 3.86, 95% CI 0.94–17.27, p = 0.061).

Given the biologically consistent immune profile and the separation observed in survival curves between the favorable B7-H3 low/CD3 high group and the remaining phenotypes in the four-group analysis, patients were dichotomized into a two-group model (B7-H3 low/CD3 high vs. all other immune phenotypes combined) to assess its potential clinical utility. Kaplan–Meier analysis demonstrated that patients with the favorable B7-H3 low/CD3 high immune phenotype had significantly improved OS and EFS compared with all remaining patients (log-rank χ² = 8.195, p = 0.004 and log-rank χ² = 7.351, p = 0.007, respectively) (**Figure 6C**). In univariable Cox proportional hazards regression, the favorable B7-H3 low/CD3 high immune phenotype was associated with a pronounced protective effect, corresponding to an approximately 10-fold lower hazard of death and about 4.5-fold lower hazard of an EFS event compared with the non-favorable group (**Table 6**). In parsimonious multivariable analyses adjusted for established clinical risk factors, including age at diagnosis, *MYCN* status, and prognostic risk classification, the B7-H3 low/CD3 high immune phenotype remained independently associated with significantly better OS and EFS.

**Table 6:** Association of the B7-H3 low/CD3 high immune phenotype with survival outcomes in malignant neuroblastic tumors in a binary immune phenotype model.

Endpoint	Model	Variable	HR	95% CI	p-value
OS	Univariable	B7-H3 low/CD3 high	0.095	0.013–0.715	**0.022**
	Multivariable (age–adjusted)	B7-H3 low/CD3 high	0.101	0.013–0.768	**0.027**
		Age ≥18 months	10.56	2.40–46.37	**0.002**
	Multivariable (*MYCN*–adjusted)	B7-H3 low/CD3 high	0.111	0.015–0.847	**0.034**
		*MYCN* amplification	3.25	1.13–9.34	**0.029**
	Multivariable (prognostic risk–adjusted)	B7-H3 low/CD3 high	0.115	0.015–0.874	**0.037**
		High risk	10.88	3.50–33.83	**<0.001**
EFS	Univariable	B7-H3 low/CD3 high	0.219	0.065–0.737	**0.014**
	Multivariable (age–adjusted)	B7-H3 low/CD3 high	0.221	0.065–0.746	**0.015**
		Age ≥18 months	5.40	1.99–14.66	**0.001**
	Multivariable (*MYCN*–adjusted)	B7-H3 low/CD3 high	0.251	0.074–0.856	**0.027**
		*MYCN* amplification	3.02	1.17–7.79	**0.022**
	Multivariable (prognostic risk–adjusted)	B7-H3 low/CD3 high	0.252	0.074–0.853	**0.027**
		High risk	5.08	2.18–11.87	**<0.001**

The non–B7-H3 low/CD3 high immune phenotype was used as the reference category. p-values were calculated using Cox proportional hazards regression. Bold values indicate statistical significance (p < 0.05). B7-H3, B7 homolog 3; CI, confidence interval; EFS, event-free survival; HR, hazard ratio; OS, overall survival.

## Discussion

During the study period, Serbia had an estimated population of approximately 1.0 million children aged 0–14 years (46), among whom an annual average of approximately 11 new cases of malignant neuroblastic tumors were diagnosed at the Department of Clinical Pathology, MCHCIS. This number falls within the range reported across European populations for these tumors (1), suggesting that patients diagnosed through this centralized pathology service may be broadly representative of national cases. The study cohort included both Serbian and non-Serbian residents treated at the two largest tertiary referral centers in Serbia, accounting for approximately four-fifths of all patients diagnosed at the Department of Clinical Pathology, MCHCIS who met the inclusion criteria during the study period. This further supports the relevance of the cohort within the national healthcare setting and suggests that it can be considered broadly population-representative.

In the absence of a standardized B7-H3 expression cut-off, we applied a dual data-driven approach that, to our knowledge, has not been previously described for B7-H3 cut-off determination. The convergence of all analyses on the same H-score reinforces the biological plausibility and supports the internal robustness of the selected threshold within our cohort. However, the data-driven nature of the approach carries a risk of overfitting and bias; therefore, the results of the present study should be considered exploratory and warrant external validation. No significant differences in B7-H3 expression were observed across clinicopathological parameters, indicating that B7-H3 expression appears largely independent of conventional prognostic factors in neuroblastic tumors. Although B7-H3 expression was associated with disease outcomes, its prognostic value as an independent marker in our cohort remained limited. Patients with low B7-H3 expression had better OS and EFS, in line with previous research on NB (13, 14, 17, 18). However, after adjustment for *MYCN* status and age at diagnosis, high B7-H3 expression showed only a trend toward inferior OS and EFS, which may reflect the limited number of events in our cohort or suggest that these clinicopathological parameters partly account for the prognostic effect of B7-H3. Moreover, after adjustment for prognostic risk classification, which incorporates disease stage, *MYCN* status, and age at diagnosis, thereby partially addressing the constraints imposed by parsimonious modeling, the prognostic relevance of B7-H3 was further attenuated, losing even a trend toward significance. In addition to accounting for the effects of these established prognostic factors, risk-group adjustment also addresses treatment-related variability, thereby reducing potential bias arising from differences in treatment protocols. Management of malignant neuroblastic tumors is inherently risk-adapted, with therapeutic strategies determined by pretreatment risk stratification, and the most substantial differences in treatment intensity occur between high-risk and non–high-risk patients (6, 22). Therefore, this classification is closely linked to treatment assignment and can be considered a proxy for treatment allocation. Importantly, the contribution of established clinicopathological factors to the prognostic relevance of B7-H3 expression may not have been fully captured in previous studies, as most did not incorporate multivariable Cox regression models to assess its independent prognostic value (13, 14, 17). Moreover, in studies where such analyses were performed, B7-H3 was evaluated within integrated immune-related models rather than as an individual prognostic marker (18).

The association between B7-H3 expression and patient prognosis across various malignant tumors is closely related to its interaction with the immune system (9–11, 40). Previous reports have highlighted the role of B7-H3 in suppressing NK-cell–mediated antitumor immunity in NB (9, 17). However, the association between B7-H3 expression and infiltration of immune cell populations beyond NK cells in NB remains incompletely characterized. To our knowledge, the relationship between tumor-cell B7-H3 expression in malignant neuroblastic tumors and infiltration of CD3^+^, CD4^+^, CD8^+^, CD20^+^, and CD68^+^ immune cells has not been systematically evaluated in patient-derived tumor samples. Given the typically heterogeneous and spatially compartmentalized distribution of immune cells in malignant neuroblastic tumors (8, 33–35), a hotspot-based approach focusing on regions with maximal immune cell density within each tumor was used to enable standardized assessment of immune infiltration and comparability across cases. We observed that high tumor-cell B7-H3 expression was associated with reduced CD8^+^ T-cell infiltration, which, although not directly validated by functional experiments, is consistent with the previously reported immunosuppressive role of B7-H3 in cancer (9–11). A similar inverse association between B7-H3 expression and CD8^+^ T-cell infiltration has been reported in esophageal cancer (11), prostate cancer (40), breast cancer (47), hepatocellular carcinoma (48), non–small cell lung cancer (49), osteosarcoma and rhabdomyosarcoma (9). In NB, the relationship between B7-H3 expression and CD8^+^ T-cell infiltration has primarily been investigated in preclinical models, which provide important mechanistic insights but may not fully recapitulate the complexity of the human tumor immune microenvironment. Pathania et al. demonstrated that microRNA miR-29 family members act as post-transcriptional regulators of B7-H3 expression, resulting in reduced expression of this immune checkpoint in NB cell lines. *In vivo*, miR-29–expressing murine NB models exhibited increased CD8^+^ T-cell infiltration, accompanied by enhanced cytotoxic potential, indirectly supporting a role of B7-H3–mediated immune modulation (17). Consistent with these findings, studies in pancreatic cancer (10), ovarian cancer (50), rhabdomyosarcoma (9), head and neck squamous cell carcinoma (51), and non–small cell lung cancer (49) have demonstrated that blockade or depletion of B7-H3 enhances CD8^+^ T-cell–mediated antitumor immunity. Moreover, it has been shown that B7-H3 may inhibit CD8^+^ T-cell function by enhancing the glycolytic activity of tumor cells in oral squamous cell carcinoma and melanoma, thereby competitively reducing glycolysis in CD8^+^ lymphocytes (52). Conversely, only a limited number of studies have reported findings suggesting a potential CD8^+^ T-cell–stimulating role of B7-H3 in certain malignancies, including pancreatic cancer, mastocytoma, and hepatocellular carcinoma. It remains unclear whether these conflicting results are mediated by engagement of distinct, as yet unidentified B7-H3 receptors, or whether they instead reflect cell-, tissue-, or tumor-specific contextual factors (10). Although no significant associations were observed between B7-H3 expression and other immune-cell subsets in our cohort, previous studies have reported inverse correlations between B7-H3 expression and CD3^+^ and CD4^+^ T-cell infiltration in some adult malignancies (10, 11). Based on our literature search, we did not identify studies evaluating the relationship between B7-H3 expression and CD3^+^ T-cell infiltration, CD4^+^ T-cell infiltration, or CD20^+^ B-cell infiltration in either clinical samples or experimental models of NB, thereby limiting direct comparison with our observations. Consistent with the findings regarding CD8^+^ T cells, Pathania et al. demonstrated that the overexpression of miR-29 family members resulted in reduced CD68^+^ macrophage infiltration in murine NB models, suggesting a potential B7-H3–mediated modulation of macrophage recruitment within the tumor microenvironment (17). Similarly, in hepatocellular carcinoma and colorectal cancer, B7-H3 tumor expression has been associated with increased CD68^+^ macrophage infiltration and a shift from M1 to M2 (tumor-promoting) macrophage polarization (53).

When assessing the prognostic impact of immune cell infiltration in malignant neuroblastic tumors, our results identified CD3 as the most robust immune marker. High intratumoral CD3^+^ T-cell infiltration was associated with significantly improved OS and EFS, consistent with previous studies (8, 43). This effect persisted after adjustment for *MYCN* amplification status, age, and prognostic risk group, suggesting that a T-cell–rich immune contexture is independently associated with more favorable survival outcomes in these tumors. In addition, increased infiltration of CD4^+^ T lymphocytes was also associated with a favorable clinical course, showing a significant improvement in EFS and a trend toward better OS. Elevated CD8^+^ T-cell infiltration demonstrated a suggestive, albeit not statistically robust, association with improved outcomes. Although several studies have shown that increased CD8^+^ T-cell infiltration in NB is associated with improved outcomes, some reports have indicated that a predominance of CD4^+^ T cells over CD8^+^ T cells correlates with better survival (8, 35). Furthermore, several studies have reported that B-cell infiltration in NB is rare; however, this observation may be influenced by the spatial organization of B cells, which is largely confined to specific tumor regions and may not always be captured in tissue sections or biopsies. Larger B-cell aggregates may be localized at the tumor periphery, forming organized lymphoid structures and follicles. Schaafsma et al. showed that B-cell infiltration is strongly associated with improved prognosis in NB, in contrast to our results, which showed no association (35). Finally, accumulating data indicate that macrophages within the NB tumor microenvironment predominantly acquire an M2-like, immunosuppressive phenotype, thereby contributing to tumor progression, immune evasion, and worse prognosis (54). The lack of an association between CD68^+^ cells and survival in our study might be attributable to the use of CD68 as a pan-macrophage marker, which does not allow discrimination between distinct macrophage polarization states.

Furthermore, we assessed the prognostic impact of an integrated immune checkpoint–immune cell model incorporating B7-H3 expression and CD3^+^ T-cell infiltration, which, to our knowledge, has not been systematically investigated in malignant neuroblastic tumors. A related integrative immune approach was reported by Zeng et al., who explored a broad panel of immune-checkpoint molecules and incorporated OX40, B7-H3, ICOS, and TIM-3 into a composite immunoscore associated with NB prognosis (18). In our study, tumors characterized by low B7-H3 expression and high CD3^+^ T-cell infiltration exhibited the most favorable outcomes, consistent with a more permissive immune contexture. In contrast, the phenotype defined by high B7-H3 expression and low CD3^+^ infiltration represented the most adverse group, with an approximately 14-fold higher hazard of death and a nearly sixfold higher hazard of an EFS event compared with the favorable phenotype. However, all estimates related to the combined model in our study, including those derived from regression analyses and C-index–based discrimination, should be interpreted with caution, given the wide confidence intervals, likely reflecting the limited number of survival events. In this context, potential overestimation of effect sizes, particularly those of large magnitude, cannot be excluded.

Model discrimination analyses suggested a possible improvement in prognostic performance with the incorporation of the combined B7-H3/CD3 phenotype compared with CD3^+^ T-cell infiltration alone, as reflected by higher C-index values for both OS and EFS. However, formal model comparison indicated only a trend toward improved model fit for OS and no statistically significant difference for EFS. This may be attributable to the relatively small number of events in our cohort, which may have limited statistical power to detect meaningful differences between models and affected the stability of the estimates.

Although the combined model did not demonstrate a statistically significant improvement in global model discrimination within our cohort, it provided meaningful refinement of risk stratification for OS within a biologically defined subgroup, as reflected by its ability to identify a particularly favorable immune phenotype not captured by CD3^+^ T-cell infiltration alone. Within the CD3-high subgroup, the incorporation of B7-H3 further refined risk stratification, as demonstrated by Kaplan–Meier analysis showing significantly improved OS in patients with the B7-H3 low/CD3 high phenotype. However, the statistical limitations of the dataset may have affected the stability of conventional Cox estimates, and the comparison between the B7-H3 low/CD3 high and B7-H3 high/CD3 high phenotypes showed a borderline association with OS. To address this, we applied Firth-penalized Cox regression, which is better suited for small-sample settings. This analysis demonstrated a statistically significant association with OS, indicating that patients with the B7-H3 low/CD3 high phenotype had an approximately sevenfold lower hazard of death compared with those with the B7-H3 high/CD3 high phenotype. Importantly, the combined model identified a subgroup with an OS of 96.6%, comparable to outcomes typically observed in low-risk NB, where OS exceeds 95% (55).

To further characterize this favorable phenotype, we applied a simplified two-group model contrasting the B7-H3 low/CD3 high phenotype against all other immune phenotypes combined. This dichotomization retained the main prognostic pattern observed in the four-group model while improving interpretability and potential clinical applicability. Patients with the favorable immune phenotype exhibited an approximately tenfold lower hazard of death and a 4.5-fold lower hazard of an EFS event (OS HR = 0.095; EFS HR = 0.219), with more pronounced effect sizes than those observed with CD3^+^ T-cell infiltration alone (OS HR = 0.27; EFS HR = 0.36). Importantly, the B7-H3 low/CD3 high immune phenotype retained a statistically significant protective effect across multivariable analyses adjusted for age at diagnosis, *MYCN* amplification status, and prognostic risk classification, providing supportive evidence of an independent association within the constraints of parsimonious modeling. These findings suggest that the prognostic value of the favorable phenotype is not merely a surrogate for established clinical risk factors but rather represents additional, immune-related biological information that, if validated in future studies, may contribute to refining existing risk stratification systems. Taken together, although the combined model did not reach the formal incremental prognostic value, the consistent direction of effect across multiple complementary analytical approaches supports the potential relevance of the combined phenotype and provides a rationale for further evaluation in larger, independent cohorts.

Beyond their potential prognostic relevance, our findings provide a rationale for further investigation of immunologically informed biomarker stratification in the context of patient selection for immunotherapy. Whether specific phenotypes, such as B7-H3 high/CD3 low, may be more likely to benefit from strategies involving B7-H3 blockade or T-cell–directed approaches, such as CD3×B7-H3 bispecific antibodies or chimeric antigen receptor (CAR) T-cell therapy (9, 10, 56, 57), remains to be determined.

Several limitations of this study should be acknowledged. Although our findings are consistent with an immunosuppressive role of B7-H3 in neuroblastic tumors, functional validation is lacking. Despite the cohort being population-representative, the retrospective design may introduce selection bias and residual confounding. In addition, minor heterogeneity in treatment protocols and clinical decision-making may have influenced the results, although this is likely mitigated by the standardized risk stratification incorporated into the analyses. Furthermore, the relatively small cohort size and the sparsity of survival events limited the complexity of multivariable analyses, necessitating parsimonious modeling with a restricted number of covariates to minimize overfitting. Although partially addressed by adjustment for prognostic risk classification, this approach cannot fully substitute for a comprehensive multivariable model. Moreover, the estimates from multivariable modeling should be interpreted with caution, as small-sample effects may contribute to wider confidence intervals and reduced precision.

In addition, the high/low classification of tumor-cell B7-H3 expression and immune-cell infiltration used in this study reflects biologically meaningful differences rather than fixed or universally applicable thresholds. The determination of the B7-H3 cut-off relied on data-driven approaches, which, despite their internal consistency, introduce a potential risk of overfitting and bias. Immune-cell infiltration was dichotomized using median values, also remaining dependent on cohort-specific distributions and may be influenced by technical and analytical factors related to immunohistochemical assessment and digital image analysis. Consequently, independent external validation is required to confirm the robustness, reproducibility, and potential clinical applicability of these thresholds and to support the development of standardized cut-off values.

In conclusion, our findings are consistent with a potential immunosuppressive role of the B7-H3 checkpoint molecule in malignant neuroblastic tumors, as reflected by its association with reduced CD8^+^ T-cell infiltration. Although high B7-H3 expression was associated with worse outcomes, its independent prognostic utility in our cohort remained limited. While CD3^+^ T-cell infiltration demonstrated prognostic significance when assessed individually, its integration with B7-H3 expression enabled more precise identification of patients with a favorable prognosis. The favorable B7-H3 low/CD3 high phenotype was associated with significantly improved survival compared with all other phenotypes combined and retained its prognostic relevance after adjustment for established clinical risk factors, providing a rationale for its further exploration as a prognostic biomarker. However, given the limited statistical power of the current cohort, these findings should be considered exploratory and warrant validation in larger, independent cohorts, particularly as the incremental prognostic value of combined B7-H3/CD3 phenotyping showed only a trend toward improved OS discrimination compared with CD3 alone.

## Data Availability

The raw data supporting the conclusions of this article will be made available by the authors, without undue reservation.
